# Conservation of the Dental Pulp*Discussion of Dr. Howe’s paper was published in December, 1919, *Items of Interest*.

**Published:** 1920-05

**Authors:** Herman Prinz


### CONSERVATION OF THE DENTAL PULP *

BY HERMAN PRINZ, D.D.S.

From Dr. Howe's paper one would take it for granted that practically every pulp can be saved. Those gentlemen who are in practice will know that this is not quite true. Dr. Howe refers to some of these cases as diseased pulps in children's teeth; many of these pulps can be conserved, and they will reconstruct themselves. Even a tooth that is fractured, as he has shown, will, on rare occasions, form a callus and union may take place. A most excellent paper on this very subject of callus formation

* Discussion of Dr. Howe's paper was published in December, 1919, *Items of Interest.*

in teeth was published about ten years ago by Dr. Wunschkeim of Vienna, and he has mentioned about fifteen cases, and Dr. Ottolengui states that Dr. Conrad has one; but, gentlemen, those are isolated cases, and it does not mean we can always practice these procedures. The pulp is a transitory organ. Its work is practically finished when the tooth is completed. From that moment on, it is a retrogressive organ; it atrophies. The defensive properties of the pulp in a developing tooth are enormous. This is exactly in conformity with every other organ, where we have growth. When it ceases, resolution of that organ will rarely take place. The pulp, after twenty or twenty-five years of age, will not reconstruct itself. The peculiar anatomical arrangement of the tissues of the pulp are strong factors against it. The pulp is enclosed in a hard unyielding wall of dentin; it can not swell, and it has no lymphatics. It can not carry away the waste products, and it does not possess collateral circulation. All those factors act strongly against resolution.

Usually pulp capping is a failure. Nevertheless, in spite of all these facts, it should be our aim to conserve the pulp when at all possible. Any of the medicaments which Dr. Howe has mentioned—zinc oxide with eugenol, zinc oxide with thymol, etc.—when carefully applied without pressure, act as protectors and not as means of exercising any special therapeutic function. The idea is to protect the pulp with a non-irritating sterile cover. Pulp tissue must be looked upon as a weak structure.

Dr. Howe has said silver nitrate will prevent the progress of decay. This is absolutely true.

I wish to mention one point he has made, and that is in regard to discoloration. While this is of little importance when temporary teeth are concerned, we know that when we apply it upon the anterior teeth in the adult the discoloration may shine through the gum tissue and our patients object to it.

Another question arises: Have we any other material which, when applied to carious surfaces, will inhibit its progress or, rather, sterilize the cavity? A fifty per cent. solution of thymol, in acetone, applied to a dentin surface which must be thoroughly dehydrated, will accomplish this purpose. We cover it with a thin, hard-setting varnish protected by a double coating.

We are especially interested in the pharmacological action of silver nitrate. I deduce from his writings that Dr. Howe emphasizes that fact that silver is the vital factor. Metals themselves do not exercise any pharmacological function. If by accident a child takes a copper penny in the mouth and swallows it, in the great majority of cases it passes through the tissues without causing any harm. In by-gone days it was rather common practice in cases where there was some obstruction in the intestines, to give the patient about two pounds of mercury. It passed through the intestinal canal without causing poisonous effects.

The pure silver metal does not exercise any therapeutic function. When we employ a metallic salt, it is the acid radical which produces the effect; the metal is merely the carrying medium of this basic factor.

If you have a four-wall cavity, and you place in the cavity silver nitrate, and immediately cover it with temporary stopping. and let your patient go—when the patient returns in two or three days the cavity is not black; it is lemon yellow, because there was no chance for the light to act on the liberated nitric acid radical. Expose the silver nitrate to light, and it will turn black, provided chlorine is present. As was known, chlorine in the form of sodium chloride is present in all the tissues of the body. The metal (silver) in silver nitrate is merely the carrying factor of its therapeutic ion—nitric acid. Let me illustrate this further. We have a compound known as collargol. It contains about eighty-six per cent. of silver in a very fine state of division, while silver nitrate contains about sixtyeight per cent. of silver. But silver nitrate is at least fifteen times stronger in its antiseptic power than the former, because the very finely powdered silver is only in a very limited degree changed to a silver albumin chloride which, in turn, is ionized.

Dr. Howe has shown beautiful specimens of deep penetration of silver nitrate into the tubuli. The albumin becomes precipitated and solidly plugs up these tubuli, and any further action of micro-organisms is cut short. The only drawback is the discoloration of the dentin.

I have my doubts as to whether you can apply this principle in the treatment of the teeth of adult patients in your practice. In using dichloramin-T for the same purpose, as I have outlined in a recent number of *Dental Cosmos,* you will get the same results minus the discoloration. In filling root-canals the important point is to prevent reinfection. The blocking of the dentinal tubuli is the only absolutely safeguard to prevent this secondary infection.

Dr. Howe made the following comment on Dr. Prinz's discussion, in which he said he thought it good practice to make an attempt to save all pulps. What I wished to show is that pulps can be conserved a great deal more often than we have been inclined to think. I know it is true that the younger the better; but at the same time I never have felt that a pulp has ended its usefulness when the tooth is formed. That is like Dr. Osler's idea of chloroforming all men after they reach sixty years of age. Every man knows that when you use a bur dentin is sensitive, and the pulp is the vital organ of the tooth. A pulp can regenerate and heal as well as any other organ. Sometimes I think it does a pulp good to bleed—it prevents stasis.

As far as the question of lymphatics is concerned, I believe Dr. Noyes claimed he had found lymphatics in pulps; but we do not need lymphatics.

In regard to the silver nitrate: Dr. Prinz does not quite comprehend me. I do not say it has a therapeutic action at all; but during the act of reduction (and you are not dealing with silver nitrate, but silver oxilate), when you reduce that, you produce instantly a high degree of sterility, and block the tubuli with a material that nature tolerates—that is, silver nitrate.

I have had the opportunity to examine some cavities which had been filled with zinc oxide and eugenol, and those youngsters have gone off and did not show up again for a long time. I have afterwards found those teeth sterile when the children came back.

Try the old preparation made according to the old formula, and it works mighty well.

